# AT2R activation reduces M1-type macrophages and promotes tregs accumulation in ischemia-reperfusion-induced acute kidney injury

**DOI:** 10.3389/fphar.2026.1796687

**Published:** 2026-04-02

**Authors:** Riyasat Ali, Tahmid Faisal, Kalyani Kulkarni, Sanket Patel, Tahir Hussain

**Affiliations:** Department of Pharmacological and Pharmaceutical Sciences, Heart and Kidney Institute, College of Pharmacy, University of Houston, Houston, TX, United States

**Keywords:** AT_2_R, C21, ischemia-reperfusion, M2 macrophage, treg cells

## Abstract

**Introduction:**

Acute kidney injury (AKI) is marked by infiltration of immune cells, particularly macrophages and T cells, and their expansion as inflammatory and anti-inflammatory mediators to create a microenvironment critical for kidney injury/repair. As the angiotensin type 2 receptor (AT_2_R) is emerging as reno-protective and anti-inflammatory, this study aimed to analyze the phenotypes of macrophages and helper T cells (CD4) in response to AT_2_R activation in ischemia-reperfusion (IR)-induced AKI.

**Methods:**

Sprague Dawley rats were subjected to 30 min IR without and with AT2R agonist C21 administration. Rats were euthanized at 2 h, 3 d, and 5 d post IR. Flow cytometry of kidney digested cells was performed to analyze kidney infiltrating immune cells. For in-vitro polarization, mouse CD4 T cells were cultured in presence of various stimulus and characterized by flow cytometry and western blot analysis.

**Results:**

On day 3, there was a massive increase in macrophage/monocytes (CD68^+^) and M1 (CD68^+^CD86^+^) cell accumulation and a modest increase in M2 (CD68^+^CD163^+^) cell accumulation. The AT2R activation reduced CD68+ and M1 cells accumulation without affecting M2. Accumulation of CD4^+^CD25^+^ cells increased on day 3, and Tregs (CD4^+^CD25^+^FoxP3^+^) and Tregs-IL-10 increased at 2 h and 3 d post-IR. The AT2R agonist C21 further increased these phenotypes at all-time points. Th-17 cell accumulation increased at early time points (2 h and 3 d) but returned to normal on day 5 and was not affected by C21 treatment. Ex vivo studies revealed that AT_2_R agonist promoted CD4^+^ cell expansion into Tregs, which was blocked by the AT_2_R antagonist PD123319, PP2A inhibitor okadaic acid, or NO synthase inhibitor L-NAME, suggesting the direct involvement of AT_2_R-PP2A-NOS pathways.

**Discussion:**

Overall, AT_2_R activation reduces M1 and promotes Tregs accumulation in IR-AKI, thus shifting the kidney microenvironment towards anti-inflammation and presents a potential mechanism to limit kidney injury and promote repair.

## Introduction

Experimental renal ischemia-reperfusion (IR) causes acute kidney injury (AKI), which is characterized by sudden kidney dysfunction and mimics the clinical events that occur during and after major surgeries, trauma, and organ transplantation (Rabb et al., 1997; Brady and Singer, 1995). Upon ischemic renal injury, the immune system plays a critical but complex role in driving both the acute inflammatory response and regenerative/reparative response, which involves neutrophils, T lymphocytes, macrophages, dendritic cells, and mast cells. Of these, macrophages and T cells, particularly CD4^+^ helper cells, represent a large portion of the immune cells that play a significant role in creating a microenvironment that drives injury/repair during the AKI process (Burne et al., 2001). During the early stages of AKI, immune cell infiltrates are composed of helper CD4^+^ T cells, including the Th17 (IL-17 producing) subset, which exert pro-inflammatory deleterious effects (Luo et al., 2016; Madhur et al., 2010). During the resolution of AKI, there is a marked decrease in pro-inflammatory Th17 cells and a coincident increase in anti-inflammatory Tregs (CD4^+^ cells expressing CD25^+^ and FoxP3^+^). Treg cells play an important role in the homeostasis of the immune system and renoprotection (Gandolfo et al., 2009; Gandolfo et al., 2010; Kinsey et al., 2010), via IL-10-mediated suppression of the innate immune response, among other mechanisms (Kinsey et al., 2009). Macrophages, which are a part of the innate response, present a complex inflammatory and anti-inflammatory phenotype (Novak and Koh, 2013). In response to signals originating from the kidney during ischemia/reperfusion, circulating monocytes and macrophages infiltrate and undergo activation into various phenotypes, which are broadly classified as inflammatory M1 and anti-inflammatory M2 subtypes. Broadly, macrophages are characterized by the expression of CD68 (pan macrophage), CD68^+^CD86^+^ (M1-type), and CD68^+^CD163^+^ (M2-type) (Rubio-Navarro et al., 2016) and release pro-inflammatory (e.g., TNFα, IL-6, IL-1β) and anti-inflammatory (IL-10) cytokines during different stages of AKI (Duffield, 2010; Li and Okusa, 2010).

The AT_2_R is a component of the protective arm of the renin angiotensin system (Steckelings et al., 2022). Numerous pharmacological and genetic studies, including ours, suggest that AT_2_R is an emerging target in renoprotection under acute (IR, LPS administration) as well as chronic pathological conditions (diabetes, obesity, and high-salt intake) (Dhande et al., 2013; Dhande et al., 2015; Patel et al., 2016; Perdiguero and Geissmann, 2016). AT_2_R-mediated protection promotes resolution of renal injury and confers improved renal function (reduced proteinuria and BUN) and preservation of tubulo-glomerular structural integrity, including preservation of the cytoskeleton and tubular cells under pathological conditions (Patel et al., 2016; Ali et al., 2021). Similar protective effects of AT_2_R have been reported in the heart and brain following ischemic injury (Altarche-Xifro et al., 2009; Curato et al., 2010; Skorska et al., 2015). While these studies support the notion that AT_2_R is an immunomodulator, a comprehensive analysis of AT_2_R-mediated shift in macrophages (M2 and M1) and T cells (Tregs and Th17), potentially altering the microenvironment to make it a basis for protecting the IR kidney remains unknown. In addition, AT_2_R-mediated signaling modulating CD4^+^ Tregs is unknown. Therefore, the present study analyzed AT2R-mediated changes in the accumulation of M2/M1 macrophages and Tregs/Th17 cells in the AKI and investigated the role of the two major AT_2_R-linked signaling molecules, PP2A and NOS, in AT_2_R-mediated Treg modulation of naïve primary splenic CD4^+^ cells.

## Materials and methods

### Animals

Male Sprague-Dawley (SD) rats (250–290 g) were purchased from Harlan Laboratories (Madison, WI). The animals were housed and acclimatized for 1 week before surgery, and food and water were available *ad libitum*. The experimental protocol used in this study was approved by the Institutional Animal Care and Use Committee (IACUC) of the University of Houston and adhered to the National Institutes of Health Guide for the Care and Use of Laboratory Animals.

### Ischemia-reperfusion injury

Animals were randomized into three groups: sham, IR, and IR + C21 (0.3 mg/kg) (a synthetic AT_2_R agonist, a gift from Vicore Pharma, Sweden). The IR and IR + C21 groups were subdivided into 2 h (hour), 3 d (day), and 5 d post-ischemia groups. C21 (0.3 mg/kg, i.p.) was administered to the IR + C21 group 2 h before surgery and then daily thereafter. The IR group received saline as vehicle. To induce IR injury, rats were anesthetized and subjected to 30-min renal bilateral IR using a previously described approach (Ali et al., 2021). Animals were euthanized 2 h, 3 d, and 5 d after reperfusion. Kidneys were collected for further analysis, as described previously (Ali et al., 2021). The sham surgeries were performed in a similar manner, except that the renal arteries were not clamped.

### Kidney cell isolation

Approximately 30–40 mg of each kidney (covering the cortex and medulla) was used to isolate kidney cells by collagenase digestion (Collagenase Type 1, 0.5 mg/mL, from Sigma United States of America) and centrifugation, as described previously (Gandolfo et al., 2009). The total kidney cells were counted and checked for viability using trypan blue.

### Kidney infiltrating treg cell analysis

For T-cell analysis, nearly half a million kidney cells were washed with staining buffer (PBS +0.1% FBS +1% sodium azide) (200 µL) and centrifuged at 500 *g* for 5 min. The cell pellet was re-suspended in 100 µL PBS and stained with fixable live/dead dye (Fixable Viability Stain 570, BD Biosciences, United States of America) for 20 min at 4 °C. The cells were washed with PBS and centrifuged at 500 *g* for 5 min. The pellet was resuspended in staining buffer (100 µL) and incubated with Fc block (CD16/CD32 monoclonal antibody, eBioscience, United States of America) and surface stained with anti-rat CD3-FITC, anti-rat CD4-APC-Cy7 antibodies, and anti-rat CD25-PE antibodies (Biolegend, United States of America) for 20 min at 4 °C in the dark. Proper isotype controls (BioLegend, United States of America) were used to minimize non-specific and background staining.

For intracellular staining, cells were resuspended in 200 µL of fixation/permeabilization solution (BD Bioscience, United States of America) for 20 min at 4 °C in the dark, washed twice with the same buffer, and centrifuged at 500 *g* for 5 min. The cells were then incubated with Alexa Fluor 647 labelled anti-rat FoxP3 (BioLegend, United States of America) and anti-rat IL-10 (BD Bioscience, United States of America) in 100 µL permeabilization/wash buffer for 30 min at 4 °C. The cells were washed twice with 200 µL permeabilization/wash buffer, resuspended in 200 µL staining buffer, and subjected to flow cytometry using a BD FACSMelody. Data were analyzed using FlowJo software.

### Kidney infiltrating macrophage analysis

Macrophage analysis was performed by quantifying CD68^+^ (PAN macrophages), CD68^+^CD86^+^ (M1 macrophages), and CD68^+^CD163^+^ (M2 macrophages) cells using anti-rat CD68-Alexa Fluor 647, anti-rat CD163-FITC (Bio-Rad, United States of America), and anti-rat CD86-Biotin (Biolegend, United States of America) antibodies and streptavidin APC (eBioscience, United States of America). Antibody staining, washing, and gating were performed as previously described (Rubio-Navarro et al., 2016). The cells were washed twice with 200 µL permeabilization/wash buffer, resuspended in 200 µL staining buffer, and then subjected to flow cytometry using a BD FACSMelody. Data were analyzed using the FlowJo software.

### PP2A enzymatic activity

PP2A enzymatic activity was assessed using the PP2A Immunoprecipitation Phosphatase Assay Kit (Millipore), according to the manufacturer’s instructions. Briefly, kidney homogenates were immunoprecipitated with a PP2Ac-specific antibody (clone1D6, Millipore). A synthetic phosphopeptide, as a substrate for PP2Ac, the catalytic subunit of PP2A, as incubated (30 °C for 10 min) with the immunoprecipitated immune complex at 30 °C for 10 min. After centrifugation, the supernatants (25 µL) were transferred to a 96-well plate, and the released phosphate was measured by adding 100 µL malachite green solution and reading at 650 nm. The phosphate concentrations were calculated using a standard curve. PP2Ac activity is expressed as phosphate release (pmol/µg protein).

### *Ex vivo* treg expansion

Male mice (10–12-week-old C57/BL6) were euthanized under isoflurane, and the spleen was collected and processed in RPMI-1640 medium (Gibco) with 10% fetal bovine serum. The spleen was minced, passed through a 40 µm strainer, and collected in RPMI-5 media. Red blood cells were lysed using ACK lysis buffer and washed with PBS and MACS buffers. The cells were resuspended in 90 µL of MACS buffer per 10 × 10^7^of total cells. The cells were magnetically labelled with anti-mouse CD4^+^ (L3T4) microbeads (10 µL of CD4 L3T4 Microbeads per 10^7^ total cells) and only the CD4^+^ T cells were separated (by positive selection) by a magnetic column using MACS buffer with 0.5% sodium azide. The T cell receptors in CD4^+^ T cells were activated using CD3 (100 ng/mL) and CD28 (1 μg/mL) specific antibodies and 1-1.5 × 10^6^ cells/well were plated in 6 well plate at 37 °C in an incubator with carbon dioxide for 4 days with different treatments as follows. The cells were treated with anti-mouse IL-2 (100 ng/mL) for T cell expansion on day 2. On day 3, we treated the cells with human TGF-β1 (5 ng/mL), the AT_2_R agonist C21 (0.1 µM), the AT_2_R antagonist PD123319 (10 µM), okadaic acid (1 µM), and/or L-NAME (1 µM). To reduce intracellular glycoprotein secretion, the cells were treated with monensin sodium on day 4. On day 5, we collected the cells and performed flow cytometry analysis using anti-mouse CD4-APC-Cy7 antibody, PE CSF 594 viability dye, Alexa Fluor 647 conjugated Isotype control, and FoxP3-Alexa Fluor 647. Flow cytometry was performed using the BD Accuri C6 flow cytometer.

### Statistical analysis

Data are presented as mean ± SEM. Statistical analysis was performed using one-way analysis of variance (ANOVA) with Fisher’s LSD test for multiple comparisons and variations between more than two groups. Statistical significance was set at p < 0.05.

## Results

### Assessment of renal injury after IR injury

We previously reported a detailed kidney function analysis in these animals. We found that IR injury reduced kidney function at different time points, as measured by plasma creatinine, blood urea nitrogen, proteinuria, and kidney injury molecule-1. However, C21 treatment preserved kidney function and improved functional recovery (Ali et al., 2021).

### AT_2_R agonist treatment increases M2/M1 macrophage ratio in the post-IR kidneys

**FIGURE 1:**
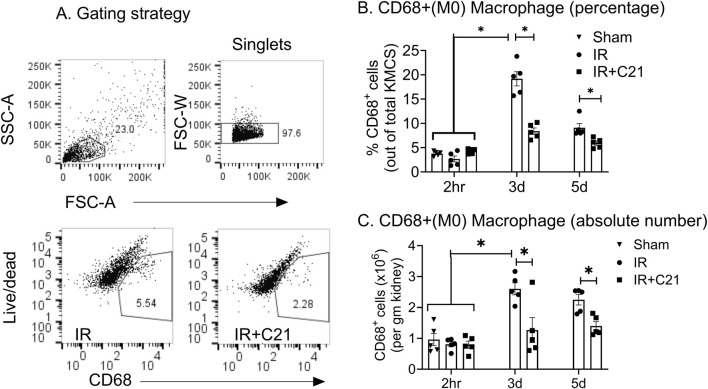
Kidney infiltrating macrophage analysis in sham, ischemia-reperfusion (IR) and IR + C21 treated SD rats. SD rats were subjected to 30 min IR, and saline or C21 were injected, and the rats were euthanized after 2 h, 3 days, and 5 days as described in the methods. Kidney cells were isolated and analyzed by flow cytometry. **(A)** Representative FSC-A/SSC-A showing the gating strategy. Pan macrophage (CD68^+^) analysis **(B)** percentage, **(C)** absolute number. Data are expressed as mean ± SEM, (n = 5), analyzed by one-way ANOVA with Fisher’s LSD test for multiple comparisons, p values less than 0.05 (p < 0.05) are considered as significant.

**FIGURE 2:**
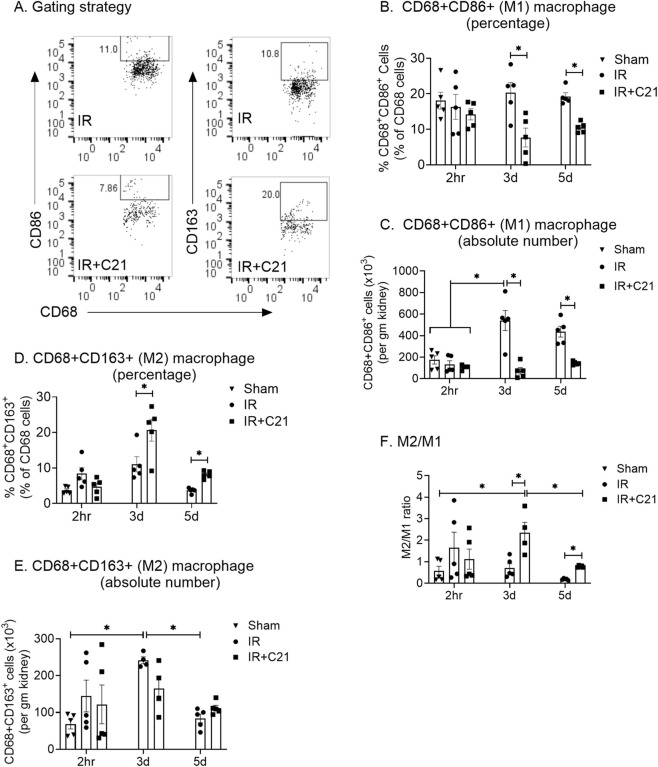
Kidney infiltrating macrophage analysis in sham, ischemia-reperfusion (IR) and IR + C21 treated SD rats. SD rats were subjected to 30 min IR, and saline or C21 were injected, and the rats were euthanized after 2 h, 3 days, and 5 days as described in the methods, kidney cells were isolated and analyzed by flow cytometry. **(A)** Representative FSC-A/SSC-A showing the gating strategy. M1 macrophage (CD68^+^ CD86^+^) analysis **(B)** percentage, **(C)** absolute number, M2 macrophage (CD68^+^ CD163^+^) **(D)** percentage, **(E)** absolute number, **(F)** ratio of M2/M1. Data are expressed as mean ± SEM, (n = 4 or 5), analyzed by one-way ANOVA with Fisher’s LSD test for multiple comparisons, p values less than 0.05 (p < 0.05) are considered as significant.

Total kidney macrophage (CD68^+^) accumulation was similar (0.96 ± 0.20 × 10^6^ to 0.81 ± 0.08 × 10^6^ cells/gm of kidney weight) in all groups at the 2 h time point. However, at 3-day, IR injury showed massive macrophage accumulation (2.6 ± 0.19 × 10^6^ cells per/gm), which was sustained for 5-day (2.25 ± 0.17 × 10^6^ cells/gm). Overall, we observed 20%–30% post-stained viability among all samples. We observed significantly higher M1 macrophages on day 3 (539.37 ± 93.41 × 10^3^ cells/gm) which was reduced on day 5 (436.85 ± 49.88 × 10^3^ cells/gm). Similarly, M2 (CD68^+^CD163^+^) macrophages showed slight increase only at 3 d (241.66 ± 9.50 × 10^3^ cells/gm) and decreased at 5 d post-IR. Interestingly, on day 3, C21 treatment significantly reduced total macrophage CD68^+^ (IR vs. IR + C21; 2.60 ± 0.19 vs. 1.27 ± 0.17 × 10^6^ cells/gm) and M1 phenotype CD68^+^CD86^+^ accumulation (IR vs. IR + C21; 539.37 ± 93.41 vs. 73.16 ± 30.29 × 10^3^ cells/gm) but had no effect on CD68^+^CD163^+^ M2 macrophages (IR vs. IR + C21; 241.66 ± 9.50 vs. 165.21 ± 33.40 × 10^3^ cells/gm). However, the M2/M1 ratio remained significantly greater (>2-fold) in C21 treated groups compared in the IR group at days 3 (IR vs. IR + C21; 0.71 vs. 2.34) and day 5 (IR vs. IR + C21; 0.19 vs. 0.78). We analyze the percentage of these cells, but we observed similar pattern with a minor shift in significance level (Figures 1, 2).

### AT_2_R agonist augments CD4^+^CD25^+^ FoxP3^+^ T cells expansion in IR injury

**FIGURE 3:**
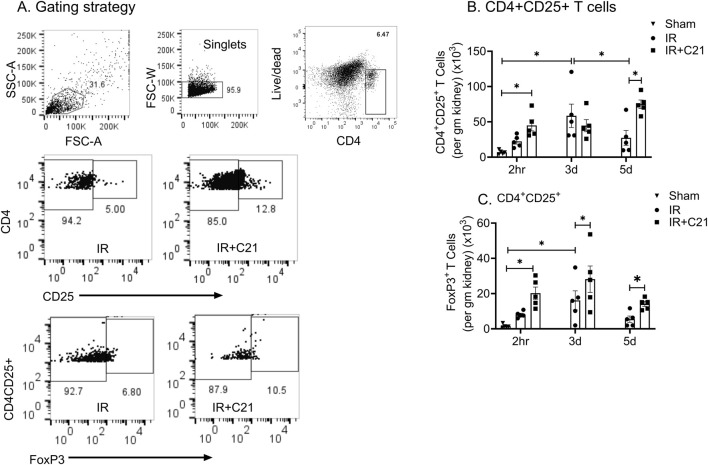
Flow cytometry analysis of kidney infiltrating CD4^+^CD25^+^ and CD4^+^CD25^+^ FoxP3^+^ T cells in sham, ischemia-reperfusion (IR) and IR + C21 treated SD rats. SD rats were subjected to 30 min IR and saline or C21 were injected, and the rats were euthanized after 2 h, 3 days, and 5 days as described in the method and kidney cells were isolated and analyzed by flow cytometry. **(A)** Representative FSC-A/SSC-A showing the gating strategy, **(B)** CD4^+^CD25^+^ T cells analysis, **(C)** CD4^+^CD25^+^FoxP3^+^ T cells analysis. Data are expressed as mean ± SEM, (n = 5), analyzed by one-way ANOVA with Fisher’s LSD test for multiple comparisons, p values less than 0.05 (p < 0.05) are considered as significant.

**FIGURE 4:**
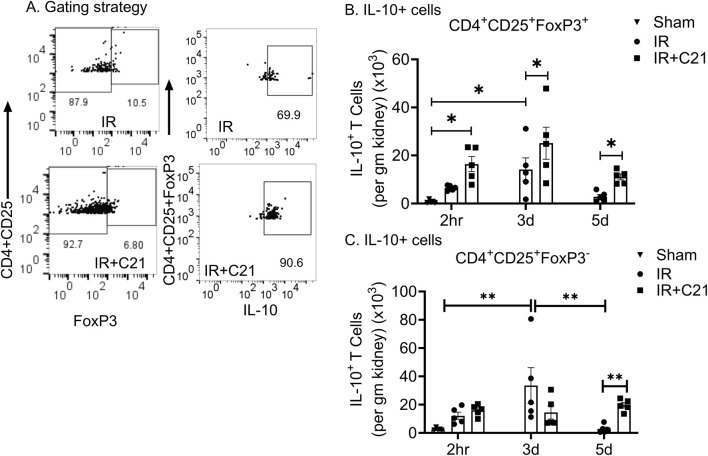
Flow cytometry analysis of IL-10 expressing CD4^+^CD25^+^FoxP3^+^ and CD4^+^CD25^+^FoxP3^-^ T cells in sham, ischemia-reperfusion (IR) and IR + C21 treated SD rats. SD rats were subjected to 30 min IR and saline or C21 were injected, and the rats were euthanized after 2 h, 3 days, and 5 days as described in the method and kidney cells were isolated and analyzed by flow cytometry. **(A)** Representative FSC-A/SSC-A showing the gating strategy, **(B)** IL-10^+^ releasing CD4^+^CD25^+^FoxP3^+^ cells analysis, **(C)** IL-10^+^ releasing CD4^+^CD25^+^FoxP3^-^ T cell analysis. Data are expressed as mean ± SEM, (n = 5), analyzed by one-way ANOVA with Fisher’s LSD test for multiple comparisons, p values less than 0.05 (p < 0.05) are considered as significant.

**FIGURE 5:**
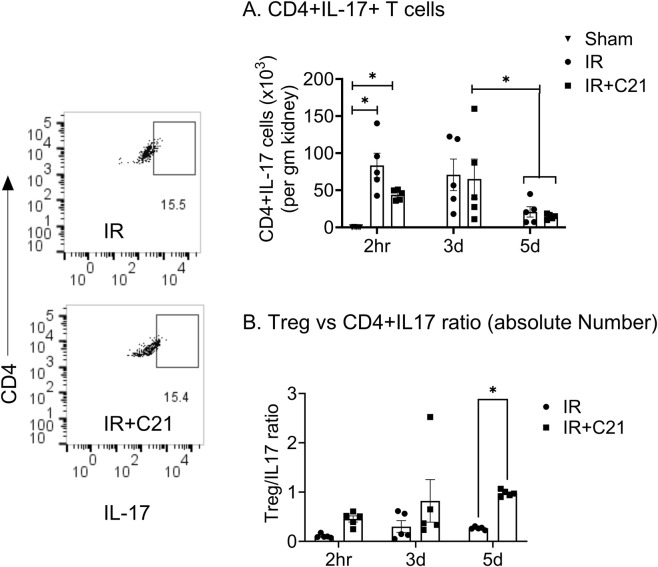
Flow cytometry analysis of IL-17 expressing CD4^+^ cells in sham, ischemia-reperfusion (IR) and IR + C21 treated SD rats. SD rats were subjected to 30 min IR and saline or C21 were injected, and the rats were euthanized after 2 h, 3 days, and 5 days as described in the method and kidney cells were isolated and analyzed by flow cytometry. **(A)** IL-17 releasing CD4^+^ cells, **(B)** ratio of Treg/CD4^+^IL-17 cells. Data are expressed as mean ± SEM, (n = 5), analyzed by one-way ANOVA with Fisher’s LSD test for multiple comparisons, p values less than 0.05 (p < 0.05) are considered as significant.

The kidney CD4^+^CD25^+^ T cell accumulation was significantly higher only at 3 d (58.53 ± 16.57 × 10^3^ cells/gm) post-IR and was similar at 2 h (22.28 ± 3.68 × 10^3^ cells/gm) and 5 d (27.30 ± 10.55 × 10^3^ cells/gm) post-IR compared to sham controls (6.94 ± 1.26 × 10^3^ cells/gm) (Figures 3A, B). However, CD4^+^CD25^+^ FoxP3^+^ Tregs and Tregs-IL-10 cell accumulation was higher at 2 h (8.08 ± 0.8, 6.51 ± 0.47 × 10^3^ cells/gm) and at 3 d (16.13 ± 5.43, 14.12 ± 4.86 × 10^3^ cells/gm) compared to the sham control (1.53 ± 0.46, 0.75 ± 0.28 × 10^3^ cells/gm) (Figure 3C). While C21 treatment did not affect CD4^+^CD25^+^ T cell accumulation at 2 h and 3 d (increased only at 5 d only), it caused a significant increase in Tregs (2 h vs. 3 d vs. 5 d; 20.20 ± 3.54 vs. 28.19 ± 7.49 vs. 14.14 ± 1.40 × 10^3^ cells/gm) (Figure 3C) and Tregs-IL-10 (2 h vs. 3 d vs. 5 d; 16.39 ± 3.12 vs. 25.12 ± 6.68 vs. 11.04 ± 1.22 × 10^3^ cells/gm) (Figures 4A, B) at all time points. In addition, IL-10 producing CD4^+^CD25^+^FoxP3^-^ cells were also remarkably increased by C21 at 5 d (IR vs. IR + C21; 2.94 ± 1.27 vs. 19.55 ± 1.89 × 10^3^ cells/gm) (Figure 4C). Similar to Tregs and Tregs-IL-10 cells, Th17 cells remarkably increased at 2 h (83.38 ± 16.59 × 10^3^ cells/gm) and 3 d (70.91 ± 21.25 × 10^3^ cells/gm) compared with sham (0.23 ± 0.10 × 10^3^ cells/gm) but were significantly reduced at 5 d (20.86 ± 6.94 × 10^3^ cells/gm) post-IR (Figure 5A). Unlike the C21 effect on Tregs/Tregs-IL-10 cells, C21 treatment did not affect Th-17 cells accumulation at any time point. However, Tregs the Th-17 ratio remained higher in the IR + C21 group (Figure 5B).

### AT_2_R agonist treatment enhances protein phosphatase 2A (PP2A) activity in IR injury

**FIGURE 6:**
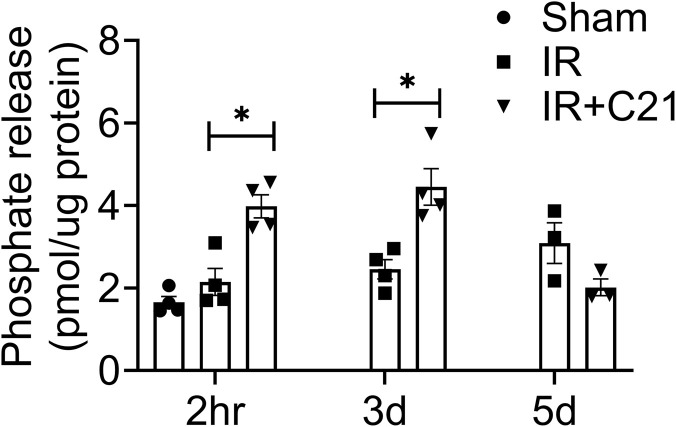
Renal PP2A activity in sham, ischemia-reperfusion (IR) and IR + C21 treated SD rats. SD rats were subjected to 30 min IR and saline or C21 were injected. The rats were euthanized after 2 h, 3 days, and 5 days and kidney cortex homogenates were prepared as described in methods and analyzed for PP2A activity. Phosphate released expressed as pmol/µg protein. Data are expressed as mean ± SEM, (n = 3-4) analyzed by one-way ANOVA with Fisher’s LSD test for multiple comparisons, p values less than 0.05 (p < 0.05) are considered as significant.

We analyzed the PP2A activity in all groups at all time-points. IR injury had no effect on PP2A activity compared to sham (sham vs. IR, 1.65 ± 0.14 vs. 2.15 ± 0.32 pmol/μg), 3 d (IR, 2.45 ± 0.23 pmol/μg), and 5 d (3.08 ± 0.44 pmol/μg). C21 treatment caused a significant increase in PP2A activity at 2 h and 3 d post-IR (IR vs. IR + C21, 2.15 ± 0.32 vs. 3.98 ± 0.28 pmol/μg), 3 d (IR vs. IR + C21, 2.45 ± 0.23 vs. 4.45 ± 0.44 pmol/μg), and had no effect on the activity at 5 d post-IR (Figure 6).

### *Ex Vivo* AT_2_R-mediated expansion of tregs via PP2A

**FIGURE 7:**
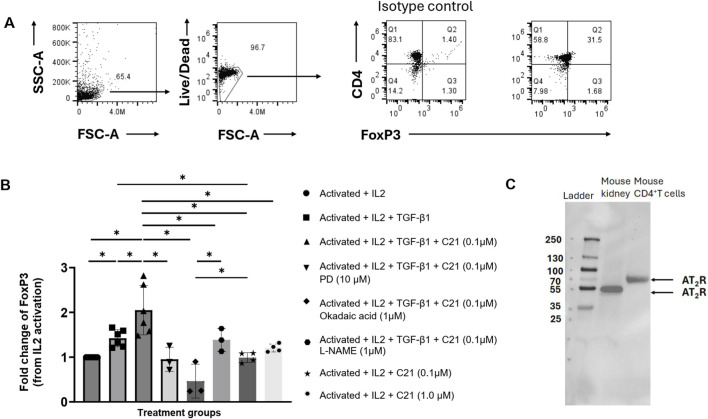
*Ex-vivo* expansion of Tregs via AT_2_R agonist C21. **(A)** Gating strategy, **(B)** fold change of FoxP3^+^ T cells from IL-2 activation of MACS sorted CD4^+^ T cells activated with anti-CD3/CD28 and treated with indicated combination of IL-2 (100 ng/mL), TGF-β1 (5 ng/mL), C21 (0.1 and 1 µM), AT_2_R blocker PD123319 (10 µM), PP2A inhibitor okadaic acid (1 µM), and NOS inhibitor L-NAME (1 µM). **(C)** Western blot showing the expression of AT_2_R in mouse CD4^+^ T cells and mouse kidney cortex homogenate as control. Data are expressed as mean ± SEM (n = 3-6). One-way ANOVA was used to analyze the data. Fisher’s least significant difference test was performed as a *post hoc* test for multiple comparisons. *p* < 0.05 was considered significant.

Splenic CD4 T cells showed AT_2_R expression as observed by Western blot analysis. As expected, IL-2 and TGF-β1 significantly expanded CD4^+^ into Tregs, which was further expanded by agonist C21. The C21-induced increase in Treg expansion was remarkably blocked by the AT_2_R antagonist PD123319 (10 μM), PP2A inhibitor okadaic acid (0.1 μM), and NO synthase inhibitor L-NAME (1 μM). While the NOS inhibitor attenuated only the C21-mediated increase in Treg levels, okadaic acid reduced Tregs to below IL-2 levels (Figure 7).

## Discussion

Renal IR-induced AKI goes through an injury phase and a repair phase marked by immune cell infiltration/accumulation in the kidney and changes in their phenotypes from inflammatory to anti-inflammatory (Burne et al., 2001; Jang and Rabb, 2015). The roles of T cells, particularly anti-inflammatory Tregs, inflammatory Th17, and macrophage phenotypes, inflammatory M1 vs. anti-inflammatory M2, are well reported (Burne et al., 2001; Gandolfo et al., 2009; Lee et al., 2024; Mehrotra et al., 2020; Wang et al., 2020; Huen and Cantley, 2015). In light of several reports that AT_2_R is an emerging target to protect against kidney injury under various pathological conditions (Fatima et al., 2023; Patel et al., 2024; Zhang et al., 2023; Patel et al., 2016; Fussi et al., 2021; Faisal and Hussain, 2025), it remains unknown how these immune cell profiles and levels in the kidney are affected by the activation of AT_2_R in AKI. The present study focused on analyzing the kidney levels of Treg vs. Th17 and M2 vs. M1 phenotypes in response to AT_2_R activation in an IR-induced AKI model. Our analysis revealed that AT_2_R activation with its agonist C21 increased Tregs and decreased M1 (CD68^+^CD86^+^) during all phases of AKI, that is, the injury, transition, and late phases, but had no effect on Th17 and M2 (CD68^+^CD163^+^) levels at any stage of AKI. As expected (Mehrotra et al., 2020), Th17 significantly increased within 2 h of IR, but decreased during the late (repair) phase. While Th17 in IR and IR + C21 remains similar, an increase in Tregs by C21 shifts the balance between Tregs and Th17 toward anti-inflammatory, as confirmed by TNFα, IL-6, and IL-10 measurements earlier (Ali et al., 2021). As Tregs can be of several types (Peterson, 2012), our analysis revealed that the accumulated Tregs are IL-10-expressing and thus anti-inflammatory and potentially suppress the immune response to cause injury (Ali et al., 2021). In addition, we observed that CD4^+^CD25^+^ cells, which induce Tregs by expressing FoxP3 (Fontenot et al., 2003), were also increased by C21 treatment during the late phase, i.e. 5 d post IR. This novel observation may have long-term beneficial effects by transforming CD4^+^CD25^+^ into Tregs. These cells can also release IL-10 independent of FoxP3 expression, as shown recently (Maynard et al., 2007). Specifically, CD4^+^CD25^+^ cells have a subset that is devoid of FoxP3, termed NO-Tregs, but releases IL-10. These Treg cells are derived from a subset of CD4^+^CD25^−^ cells by nitric oxide induction independent of cGMP but depends on p53, IL-2, and OX40 axis (Niedbala et al., 2007). Moreover, AT_2_R via phosphatases particularly PP2A, and NO-release might enhance both types of Tregs, i.e., Tregs and NO-Tregs. Our findings also supported this notion in that CD4^+^CD25^+^FoxP3^-^ express IL-10, which likely contributes to the C21-induced anti-inflammation. However, detailed study needs to understand the precise signaling events happen during AT_2_R-mediated signaling. Overall, AT_2_R activation skews toward CD4^+^CD25^+^ towards IL-10 producing Tregs (both FoxP^+^ and FoxP3^-^) without affecting Th17 levels, thereby contributing to the anti-inflammatory microenvironment in the injured kidney and protecting it from functional and structural damage during AKI.

This shift in the kidney microenvironment may also have been caused by changes in the macrophage phenotypes. Macrophage phenotypes are far more complex (Lee et al., 2011; Lech et al., 2014), as M1 phenotype is characterized by production of IL-12, IL-23, nitric oxide (NO) and reactive oxygen species (Verreck et al., 2004) while M2 showed the ‘resting’ phenotype and characterized by expression of CD163, CCR2, CXCR1, and CXCR2 and release anti-inflammatory molecules such as IL-4, and IL-10 (Martinez et al., 2009) but for simplicity, we use the terms M2 and M1, which were measured by the limited markers (such as CD68, CD86 and CD163 surface expression) in this study. However, macrophage heterogeneity and functional plasticity need detailed analysis of surface markers as well as the inducible markers such as NO, arginase-1 or CD206. Our assessment in this study gives basic understanding of macrophage roles in IR-mediated AKI. We observed the C21 treatment completely suppressed inflammatory M1 accumulation and had no effect on anti-inflammatory M2. In light of our earlier report that C21 treatment reduces the chemoattractant MCP-1 levels in the kidney (Ali et al., 2021; Patel et al., 2019), it is reasonable to speculate that such a reduction in MCP-1 levels is likely responsible for inhibiting macrophage/monocyte (CD68^+^) recruitment to the IR kidney, thus reducing M1 accumulation. However, possibilities of other events such apoptosis and efferocytosis need to be studied to understand the overall macrophage dynamics in the event of pathological conditions such as ischemia, infections, and inflammation. The source of M2 increased, equally in IR and IR + C21, in the kidney could be the resident macrophages, as the number of M2 (150x10^3^–250x10^3^) was lower than the resident numbers of CD68^+^ (1x10^6^) (Figure 1) (Patel et al., 2019; Perdiguero and Geissmann, 2016). However, the Tregs that accumulate earlier in the C21-treated group and are known to increase M2 (Liu et al., 2011; Cai et al., 2021) and limit M1(Taams et al., 2005; Panduro et al., 2018) transition may, in part, be responsible for affecting the macrophage profile. As expected, M2 levels during the middle phase, which is the early point of the reparative phase, increased, but were similar in the IR and IR + C21 groups. We (Dhande et al., 2015) and others (Zheng et al., 2025) have shown by *ex vivo* studies that AT_2_R activation skews macrophages towards the M2 phenotype (decreases M1 and increases M2) under LPS-induced macrophage activation. In this animal model, we did not observe an increase in M2 macrophages following C21 treatment. Whether the LPS vs. IR model or the *ex vivo* vs. *in vivo* model creates this discrepancy is not clear. In addition, the net tissue accumulation of immune cells depends on the balance of bidirectional trafficking in injured organs (Wilson et al., 2010; Banks et al., 2012). Therefore, it is possible that AT_2_R activation causes an M2 increase in the present study, but their net accumulation in the IR kidney remained same, while they might still contribute to the dynamic anti-inflammatory/reparative microenvironment. Overall, it is clear that C21 treatment increased the M2/M1 ratio and likely contributed to the anti-inflammatory effects, repair, and renoprotection against IR-induced AKI.

Unlike many GPCR’s, AT_2_R is linked to phosphatases, particularly PP2A (Steckelings et al., 2022; Peluso et al., 2018). In our animal studies, we observed in C21+IR kidneys an enhanced PP2A activity, which is associated with increased levels of Tregs. A recent study reported the important role of PP2A in Treg modulation (Apostolidis et al., 2016). However, kidney samples present not only immune cells, but most of the cells are kidney cells, such as epithelial cells. Therefore, it could be argued that the increase in PP2A activity in the kidney cortex homogenate does not necessarily represent enhanced activity in CD4^+^/Tregs and is associated with their modulation. To address the role of AT_2_R and PP2A pathways in Treg modulation, we employed splenic CD4^+^ cells and cultured them as primary cells. We observed that AT_2_R agonist C21 treatment caused a significant increase in Treg modulation stimulated by IL-2 and TGF-β1, which are required for Treg expansion (Horwitz et al., 2008). C21-induced Treg modulation was blocked by the AT_2_R antagonist PD123319, the PP2A inhibitor okadaic acid, and the NOS inhibitor L-NAME, suggesting the involvement of the AT_2_R-PP2A-NOS pathway. Both enzymes are linked to AT_2_R and mediate their responses in a number of cell types (Steckelings et al., 2022). In particular, the role of these signaling pathways has been elucidated in AT_2_R-mediated sodium transport in kidney epithelial cells and natriuresis (Kemp et al., 2014; Hakam et al., 2006). In addition, we reported the role of NOS in the AT_2_R-mediated release of IL-10 in macrophages and kidney epithelial cells (Dhande et al., 2013; Dhande et al., 2015). In this study, it is interesting to note that the NOS inhibitor attenuated only the AT_2_R-mediated increase in Tregs, but okadaic acid lowered Tregs below the levels stimulated by IL-2. This could be explained by the critical roles of IL-2 signaling and PP2A in the expansion and stability of Tregs (Sharabi et al., 2019). However, it is known that IL2 is essential for the development and function of Tregs, and PP2A is highly active in Tregs and is critical for their functions. The PP2A inhibitor okadaic acid enhances the phosphorylation of downstream targets of IL2 signaling (e.g., AKT and mTOR), which can destabilize Tregs and shift them toward an effector phenotype (Sharabi et al., 2019; Khan et al., 2021). These studies clearly support the direct role the AT_2_R in modulating and expanding CD4^+^ into Tregs via PP2A and NOS stimulation.

In summary, we report that AT_2_R activation shifts the microenvironment towards anti-inflammation by increasing Treg accumulation and simultaneously decreasing M1 accumulation during IR-AKI. A direct role of AT_2_R activation in Treg formation via the PP2A-NOS pathway was also revealed. Interestingly, AT_2_R activation does not affect the accumulation of M2 and Th17 cells in IR-AKI, while the role of AT_2_R in skewing towards M2 types in *ex vivo* studies has been reported (Dhande et al., 2015). Thus, these findings also indicate that the observation made in the *ex vivo* studies for immune cell modulation may not necessarily reflect in the organ accumulation of these cells. As accumulation is regulated by factors, such as bidirectional movement of cells in the affected organs and their stability, even though the modulation might have occurred. Overall, the AT_2_R-mediated anti-inflammatory microenvironment via discrete immune cell modulation/accumulation forms the basis of its renoprotective activity during the early and late phases of AKI.

## Data Availability

The raw data supporting the conclusions of this article will be made available by the authors, without undue reservation.
